# Opportunities and Challenges of Brain-on-a-Chip Interfaces

**DOI:** 10.34133/cbsystems.0287

**Published:** 2025-06-17

**Authors:** Wenwei Shao, Weiwei Meng, Jiachen Zuo, Xiaohong Li, Dong Ming

**Affiliations:** ^1^ Academy of Medical Engineering and Translational Medicine, Tianjin University, Tianjin 300072, China.; ^2^ Haihe Laboratory of Brain-Computer Interaction and Human-Machine Integration, Tianjin University, Tianjin 300072, China.; ^3^ State Key Laboratory of Advanced Medical Materials and Devices, Tianjin University, Tianjin 300072, China.

## Abstract

The convergence of life sciences and information technology is driving a new wave of scientific and technological innovation, with brain-on-a-chip interfaces (BoCIs) emerging as a prominent area of focus in the brain–computer interface field. BoCIs aim to create an interactive bridge between lab-grown brains and the external environment, utilizing advanced encoding and decoding technologies alongside electrodes. Unlike classical brain–computer interfaces that rely on human or animal brains, BoCIs employ lab-grown brains, offering greater experimental controllability and scalability. Central to this innovation is the advancement of stem cell and microelectrode array technologies, which facilitate the development of neuro-electrode hybrid structures to ensure effective signal transmission in lab-grown brains. Furthermore, the evolution of BoCI systems depends on a range of stimulation strategies and novel decoding algorithms, including artificial-intelligence-driven methods, which has expanded BoCI applications to pattern recognition and robotic control. Biological neural networks inherently grant BoCI systems neuro-inspired computational properties—such as ultralow energy consumption and dynamic plasticity—that surpass those of conventional artificial intelligence. Functionally, BoCIs offer a novel framework for hybrid intelligence, merging the cognitive capabilities of biological systems (e.g., learning and memory) with the computational efficiency of machines. However, critical challenges span 4 domains: optimizing neural maturation and functional regionalization, engineering high-fidelity bioelectronic interfaces for robust signal transduction, enhancing adaptive neuroplasticity mechanisms in lab-grown brains, and achieving biophysically coherent integration with artificial intelligence architectures. Addressing these limitations could offer insights into emergent intelligence while enabling next-generation biocomputing solutions.

## Introduction

Brain–computer interface (BCI) technology represents a transformative innovation that enables direct communication between the human brain and a computer or external devices. By detecting neural activity through electrodes, BCIs bypass traditional motor functions, allowing users to control devices through acquisition, manipulation, analysis, and translation of brain signals. In healthcare, BCIs offer immense potential for patients with neurological disorders, including those with paralysis, spinal cord injuries, and conditions like amyotrophic lateral sclerosis [1–3]. Nevertheless, various constraints on in vivo brain research, encompassing ethical considerations and limitations in intervention, continue to impede the comprehensive analysis of brain intelligence and information interaction using BCI technology. The development of stem cell differentiation and cell culture techniques in vitro has facilitated the prolonged sustenance and maturation of biological neural networks in the laboratory. These in vitro “lab-grown brains” hold the potential to overcome the limitations of the BCI technologies described above.

Consequently, in 2008, the concept of “brain-on-a-chip” emerged [4], referring to a biological neural network integrated into an engineered system capable of recording electrophysiological activity via microelectrodes and exhibiting reliable computational functions. Importantly, it has been demonstrated that lab-grown brains exhibit diverse complex electrical activities, demonstrating network plasticity similar to that observed in human and animal brains [5]. Although the initial definition emphasized the crucial role of electrical signals in brain-on-a-chip systems, subsequent research has increasingly regarded the brain-on-a-chip as a type of organ-on-a-chip [6]. This perspective involves the use of bioengineering techniques, such as microfluidics, to culture and design neuronal cells, thereby enabling the in vitro simulation of the brain [7]. In recent years, with the advancement of electrode technologies [8], particularly the emergence of microelectrode arrays (MEAs), the electrophysiological response characteristics, especially network activity, of lab-grown brains under stimulus input have garnered increasing attention. Currently, aided by advanced stimulation and recording techniques, brain-on-a-chip systems can establish an interface between lab-grown brains and computers. Furthermore, the computational capabilities of lab-grown brains enable the accomplishment of specific tasks such as spoken digit classification [9,10], image recognition [11], and robotic control [12]. Therefore, the concept of a brain-on-a-chip has partially reverted to its original definition. However, given the previous conceptual adaptations, the term “brain-on-a-chip” is no longer sufficient to encompass research on lab-grown brains in the context of biocomputing and information interaction. Drawing inspiration from BCI, we introduce the concept of a brain-on-a-chip interface” (BoCI), designed to encode and decode the electrophysiological activities of lab-grown brains and facilitate the interaction between a biological neural network and the external environment through stimulus–feedback mechanisms.

Compared to other brain-inspired technologies such as BCIs, digital twin brains, and neuromorphic computing, BoCIs present fundamental differences and notable advantages. The lab-grown brain in a BoCI system refers to dissociated neurons or brain slices obtained from rodent species, as well as 2-dimensional (2D) neural networks and 3-dimensional (3D) brain organoids differentiated from embryonic stem cells or induced pluripotent stem cells. However, BCIs target the brains of humans or animals in vivo. Despite this difference, both systems share a fundamental similarity in utilizing electrodes as intermediaries, employing encoding and decoding technologies to enable neural signal acquisition and external information input. In terms of application, BCI technology has already been successfully implemented in fields such as medical rehabilitation, robotics, gaming, and neuroscience. BoCIs, however, are still in the early stages of development. Compared to traditional BCIs, BoCI systems exhibit superior plasticity and scalability due to their compatibility with engineered neural networks. Furthermore, BoCIs bypass the ethical controversies associated with invasive human or animal trials by utilizing in vitro brain models. Digital twin brains use mathematical models or artificial intelligence (AI) to simulate the structure and function of an individual’s brain [13]. Essentially, they are a virtual modeling and personalized simulation tool that does not directly communicate with the real brain. Neuromorphic computing refers to hardware or algorithms that mimic the structure and dynamic properties of biological neurons and synapses [14]. Essentially, it is a computational architecture designed to process neural-style information more efficiently and can also serve as a backend processing module for BCI systems [15]. The BoCI system uses lab-grown brains and, essentially, aims to develop and harness biological intelligence. From the perspective of interaction with in vitro brains, there is strong reason to believe that BoCIs hold substantial potential in areas such as hybrid intelligence, autonomous control, and neuroscience.

BoCIs are considered an effective bioengineering model for fundamental science and clinical or neuropharmacological research. Advances in BoCI system construction technologies have substantially improved their structural and functional performance. From a biotechnology perspective, the complexity of neuronal networks has increased, transitioning from 2D to 3D spatial structures. The use of human-derived neurons, as opposed to rodent-derived ones, further enhances the simulation of the human brain. From an engineering standpoint, the evolution from low-density to high-density electrodes has enabled high spatiotemporal precision in the detection of electrical activity, providing more accurate and extensive data for analyzing neuronal activity. Through the development of encoding and decoding technologies, some studies have reported the computational capabilities of biological neural networks in the BoCI system [10,16,17]. This phenomenon offers new research strategies for analyzing neural activity patterns, exploring neural computation mechanisms, and developing novel intelligent technologies.

Although this emerging BoCI system is presently in its embryonic stage of development, a multitude of researchers have initiated pertinent studies, yielding compelling outcomes. This review begins by categorizing and delineating the characteristics of BoCIs. Subsequently, it discusses the interaction methods applied to lab-grown brains, followed by an exploration of hybrid intelligence research based on BoCIs. Finally, primary challenges are addressed and future development trends are outlined in the context of the prevailing research landscape.

## Interfaces between Lab-Grown Brains and Computers

**Fig. 1.**
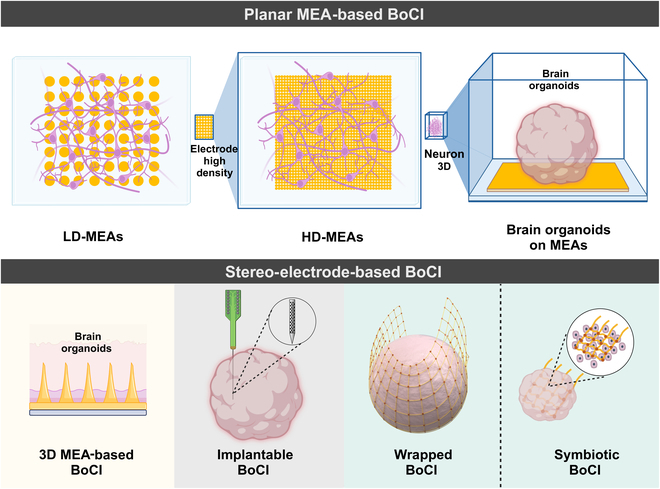
Different types of brain-on-a-chip interfaces (BoCIs). The various types of BoCI are illustrated. The various types of BoCIs are introduced, including planar and stereo-electrode-based methods. The former primarily relies on low-density or high-density planar microelectrode arrays (MEAs), while the latter employs techniques such as 3-dimensional (3D) MEAs, implantable probes, and symbiotic electrodes to capture signals from different layers and regions of brain organoids. LD-MEAs, low-density MEAs; HD-MEAs, high-density MEAs.

Lab-grown brains lack external input of information and motor behavior feedback compared to brains in vivo. Therefore, there is a critical need to develop an interactive interface facilitating communication between lab-grown brains and the external environment. The integration of electrode chips with lab-grown brains to form BoCIs serves a dual purpose: enabling the input of external information into the lab-grown brains and recording the output of the neural network. Based on the signal detection and acquisition methods used in BoCIs, 2 primary methodologies emerge: planar MEA-based BoCI and stereo-electrode-based BoCIs (Fig. 1).

### Planar MEA-based BoCIs

The use of MEAs forms the basis of BoCI reliance on 2D signal detection mechanisms derived from biological neural structures. MEAs offer noninvasive means to detect and record extracellular electrophysiological activities across multiple neuronal sites, including high-frequency action potentials and low-frequency local field potentials [18,19]. Low-density MEAs typically incorporate between 64 and 256 microelectrodes, facilitating the analysis of network-level electrophysiological phenomena. However, their spatial resolution is limited due to the substantial inter-electrode spacing, typically exceeding 100 μm, which poses challenges for single-cell electrophysiological investigations. In response to these limitations, high-density MEAs (HD-MEAs) have been developed, leveraging complementary metal–oxide semiconductor (CMOS) technology to enable precise analysis of electrophysiological signals within neuronal networks at various spatial scales [20,21]. Nowadays, commercial HD-MEAs boast an impressive microelectrode count of up to 26,400 with a pitch of 17.5 μm. Furthermore, recent advancements have yielded CMOS-based HD-MEAs with even higher microelectrode densities, featuring pitches as small as 0.25μm [22]. With the high density of microelectrodes, relatively minute electrical signals generated by axons and proximal dendrites can be detected [20,23]. These signals serve as invaluable resources for endeavors such as single-cell identification, reconstruction, and analysis of single-synapse transmission within the network [23–25]. While MEAs were initially used to detect electrophysiological activities in 2D planar neuronal networks, it is important to acknowledge the necessity of extending electronic analysis to encompass 3D neural network; 2D MEAs enable minimally invasive, long-term electrical measurements of brain organoids by directly plating them on the MEA surfaces.

### Stereo-electrode-based BoCIs

Brain organoids present a distinctive advantage in lab-grown brains simulation due to their 3D structure, albeit posing challenges in recording electrophysiological activity. Traditional planar MEA detection for brain organoids captures signals only from limited regions proximal to the contact surface, thus failing to detect activity from larger areas or deeper layers. In contrast to the direct affixation of neurons onto MEAs in a 2D cultured neural network, the integration of brain organoids into the MEA system necessitates their direct placement onto the array. This approach limits the MEAs to recording signals from only a small portion of the contact area on the spherical and planar surfaces. Consequently, there is an urgent need for the development of advanced recording technologies capable of facilitating 3D, high-resolution, large-scale electrophysiological measurements within intact organoids. In response to the requirement for stereo signal collection in brain organoids and the limitations inherent to planar signal detection, several research teams have developed various BoCIs capable of long-term coupling with brain organoids for stereo signal detection. These interfaces can be categorized into 4 types: 3D MEA-based BoCIs, implantable BoCIs, wrapped BoCIs, and symbiotic BoCIs (Fig. 1).

Three-dimensional MEA-based BoCIs feature protruding electrodes that extend into 3D space, enhancing the interface between cells with electrodes. These protrusions, with a penetration depth ranging from 40 to 100 μm, increase the effective electrode surface area, rendering them particularly adept for interfacing with 3D tissues [26,27]. Apart from detecting signals on the surface, they exhibit the capability to detect shallow internal signals. The localized curvature of these protrusions corresponds to the inherent curvature of cell membranes, facilitating the spontaneous wrapping of cells around these structures and thereby augmenting signal detection efficiency.

Implantable BoCIs, in contrast, utilize probe electrodes designed for implantation. Initially devised for invasive BCIs to monitor signals within the brain, these interfaces usually comprise one or multiple planar electrodes mounted on an implantable shaft. The design aims to maximize the number of recording sites while minimizing tissue damage during insertion. Unlike planar MEAs, implantable BoCIs can detect electrophysiological activity within brain organoids but may cause mechanical damage [28,29].

The wrapped BoCI system incorporates 2 primary types of microelectrodes: 3D basketlike MEAs and shell MEAs. Yang et al. employed 3D basketlike electrodes to facilitate the long-term suspended cultivation of brain organoids. This platform supports long-term suspended cultivation and recording while preserving the integrity of the organoids’ tissue morphology, cellular structure, and cellular composition [30]. Additionally, another 3D basketlike BoCI system was developed utilizing a liquid metal–polymer conductor (MPC)-based mesh neuro-interface, which was specifically coupled with hippocampal organoids [31]. These MPC electrodes exhibit flexibility and enhanced stretchability, allowing them to conform more effectively to the 3D shape of the brain organoid. They are capable of capturing neural signals from the spherical surface of the 3D structure and offer improved biocompatibility. Inspired by electroencephalography caps, shell MEAs were designed as soft 3D shells that enclose brain organoids, incorporating microelectrodes for electrophysiological measurements. The folding of polymer petals, guided by mechanical simulations, allows these arrays to adapt to differently sized organoids [32]. Park et al. further advanced this concept by designing a shell electrode capable of accommodating brain organoids between 480 and 600 μm in diameter, adapting to their growth and morphological changes. This shell MEA integrates multiple components for diverse signal recordings, which considerably expands experimental scope and controllability [33,34].

Symbiotic BoCIs enable noninvasive, long-term electrophysiological recording within brain organoids. McDonald et al. [35] designed a grid electrode containing 61 low-impedance TiN microelectrodes. Upon placement on the rigid grid electrode, the brain organoids gradually envelop the filaments, facilitating continuous growth. In contrast, Le Floch et al. [36] designed flexible, stretchable grid-based nanoelectronics. This kind of flexible symbiotic electrodes can be integrated with the brain organoids prior to maturation and remain embedded throughout their developmental trajectory. Their flexibility accommodates dynamic morphological changes during development, avoiding mechanical constraints.

## Information Interaction Technologies Based on BoCIs

The biocomputing ability of BoCI systems is not solely reliant on hardware systems grounded in lab-grown brain technology; rather, they are primarily contingent upon the interaction between biological networks and computers. Stimulation signals to lab-grown brains are encoded by computers, while the electrophysiological signals from lab-grown brains are transmitted to the computer for decoding. This dynamic interaction, facilitated by advanced information transmission technologies, plays a critical role in driving the development and applications of BoCI systems.

### Stimulation technologies applied in BoCI systems

**Fig. 2.**
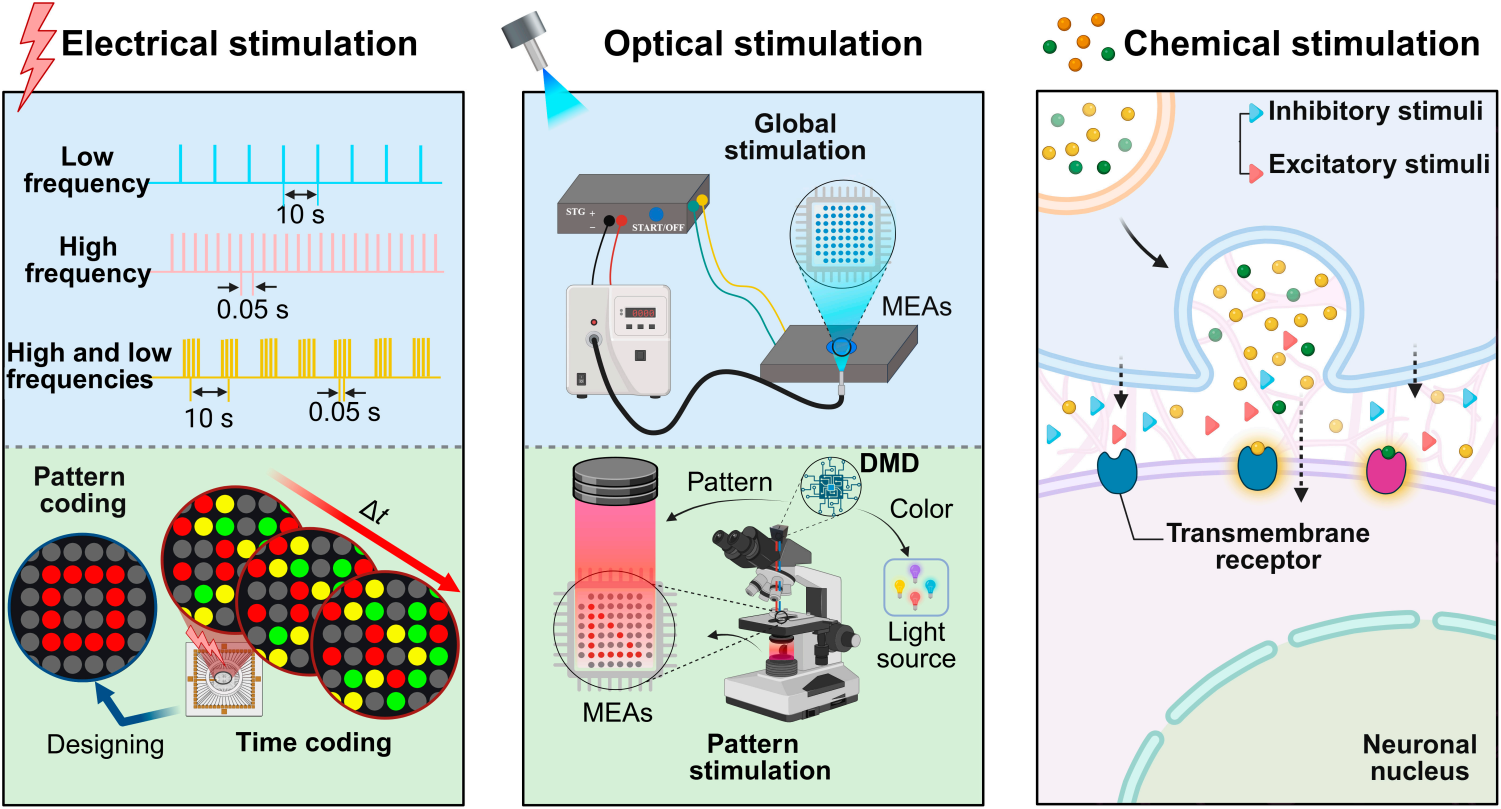
Schematic diagram of different stimulations for BoCIs. Three commonly used electrical stimulus coding modes are frequency coding, time coding, and pattern coding. Optical stimulation coding approaches include global stimulation and pattern stimulation. Chemical stimulus coding can be categorized into inhibitory stimuli and excitatory stimuli based on their efficacy. DMD, digital micromirror device.

Three primary technologies are employed to stimulate or regulate lab-grown brain systems: electrical stimulation, optical stimulation, and chemical stimulation. Electrical stimulation can be encoded through amplitude, frequency, or spatial patterns (Fig. 2). The neuronal response to electrical stimulation consists of 2 distinct phases: an initial artifact is followed by a direct, short-latency triggering response (320 ms poststimulation), likely mediated by electrical effects rather than synaptic activity. This is succeeded by a synchronized network-wide burst, peaking between 20 and 200 ms poststimulation [37–40]. Optical stimulation, in contrast, requires the introduction of photosensitive proteins into neurons via optogenetic techniques. Unlike electrical stimulation, optical approaches avoid electrical stimulation artifacts during signal acquisition, when spatially targeted illumination is applied, like digital micromirror devices. It is noteworthy, however, that optrode-based systems may introduce photoelectric or substrate-dependent noise, which can be mitigated through optrode design optimization (e.g., combination of heavy doping and electrochemical modification or internal grounded shielding layer) [41–43]. The latency period for optical stimulation is longer, ranging from 50 to 200 ms, with subsequent synchronized network bursts occurring within 100 to 300 ms and recovery to baseline taking 300 to 500 ms (Fig. 2) [37]. Chemical stimulation involves modulating neuronal activity through neurotransmitter receptor agonists or antagonists (Fig. 2). Electrical stimulation is preferred for fixed spatial targeting, whereas optical stimulation offers greater flexibility in spatial and temporal patterning, enabling more intricate control of neuronal networks [37,44–46]. While global chemical stimulation can alter the overall activity state of the network, it lacks spatial specificity. Local chemical stimulation, however, provides higher precision but requires advanced technical expertise [12,47]. Due to the variability of chemical stimulation being less predictable than electrical and optical stimulation, fewer studies have used this method as an input method for BoCI systems. Therefore, the following discussion focuses on electrical and optical stimulation.

#### Electrical stimulation

Electrical stimulation not only promotes the differentiation and maturation of brain organoids [48] but also serves as a key tool for investigating the learning and memory capacities of in vitro neuronal networks. Learning involves acquiring and regulating responses to stimuli, while memory pertains to information storage and recall [49,50]. Le Feber et al. demonstrated that low-frequency (0.2-Hz) repetitive electrical stimulation applied to cortical neuronal networks enables the encoding of parallel memory information. This finding is based on the principle of a delicate balance between network activity and connectivity, wherein novel input triggers distinct response patterns, altering synaptic connection weights, while repeated stimuli maintain equilibrium [51]. Similarly, Yang et al. showed that both 0.2- and 1-Hz frequencies activate learning and memory functions in hippocampal neurons. At 1 Hz, enhanced bursting discharge patterns and prolonged (>5-h) neuronal correlations established a robust in vitro model [50]. In addition to low-frequency stimulation, high-frequency (10-Hz) paired electrical stimulation has been used to induce long-term potentiation (LTP) and long-term depression (LTD) in cortical neurons, resulting in substantial network remodeling linked to global neuronal activity [52]. Furthermore, Chiappalone et al. [53] demonstrated that tetanic bursts (20 Hz) combined with low-frequency stimulation optimally induced plasticity and elicited enduring enhancement effects. Poli and Massobrio [54] extended these findings, employing similar stimulation paradigms to reveal robust remodeling of functional connectivity through direct stimulation.

#### Optical stimulation

In addition to electrical stimulation, researchers have employed optical stimulation to investigate the learning potential of lab-grown brains. Sumi et al. [9] utilized optogenetics and calcium imaging within a reservoir computing framework, highlighting enhanced computational capabilities. Bayat et al. [55] integrated optogenetics with MEA recordings, achieving precise control over hippocampal networks expressing channelrhodopsin-2 (ChR2) for open- and closed-loop tasks, including studies on synaptic plasticity (spike-timing dependent plasticity [STDP]). Hwang et al. [56] developed a co-culture platform with neurons and astrocytes expressing ChR2 to explore astrocytic influences on neuronal dynamics. Additional studies demonstrated timing-dependent LTP and timing-dependent LTD mechanisms and unveiled spatiotemporal processing in cortical microcircuits, advancing neural information processing research. Additional studies demonstrated STDP-based timing-dependent LTP and timing-dependent LTD mechanisms while also revealing short-term spatiotemporal memory capabilities within cortical microcircuits—advancing our understanding of neural information processing [44,57].

### Functional connectivity and plasticity of biological neural networks in BoCI systems

Understanding neural plasticity is vital for optimizing lab-grown brain systems, as it underpins learning, memory, and the adaptability of neural networks [46,58,59]. Plasticity mechanisms, such as Hebbian learning, synaptic plasticity, and neural network homeostatic plasticity, contribute to the establishment and functionality of the nervous system, forming the foundation of biological intelligence [60]. Electrophysiological data allow researchers to construct functional network topologies and analyze connectivity changes over time. Studies show that neurons self-organize into complex networks, transitioning from random to small-world configurations as they mature [61]. Chiappalone et al. [62] demonstrated increased network nodes and stronger connections in cortical cultures over 5 weeks, while Newman [63] reported heightened connectivity and modularity in early development. At the same time, Fonseca et al. [64] observed the formation of modular structures that enhance information transfer. In brain organoids, similar trends emerge, with increased complexity, neuronal firing strength, and synchrony paralleling changes [65].

**Fig. 3.**
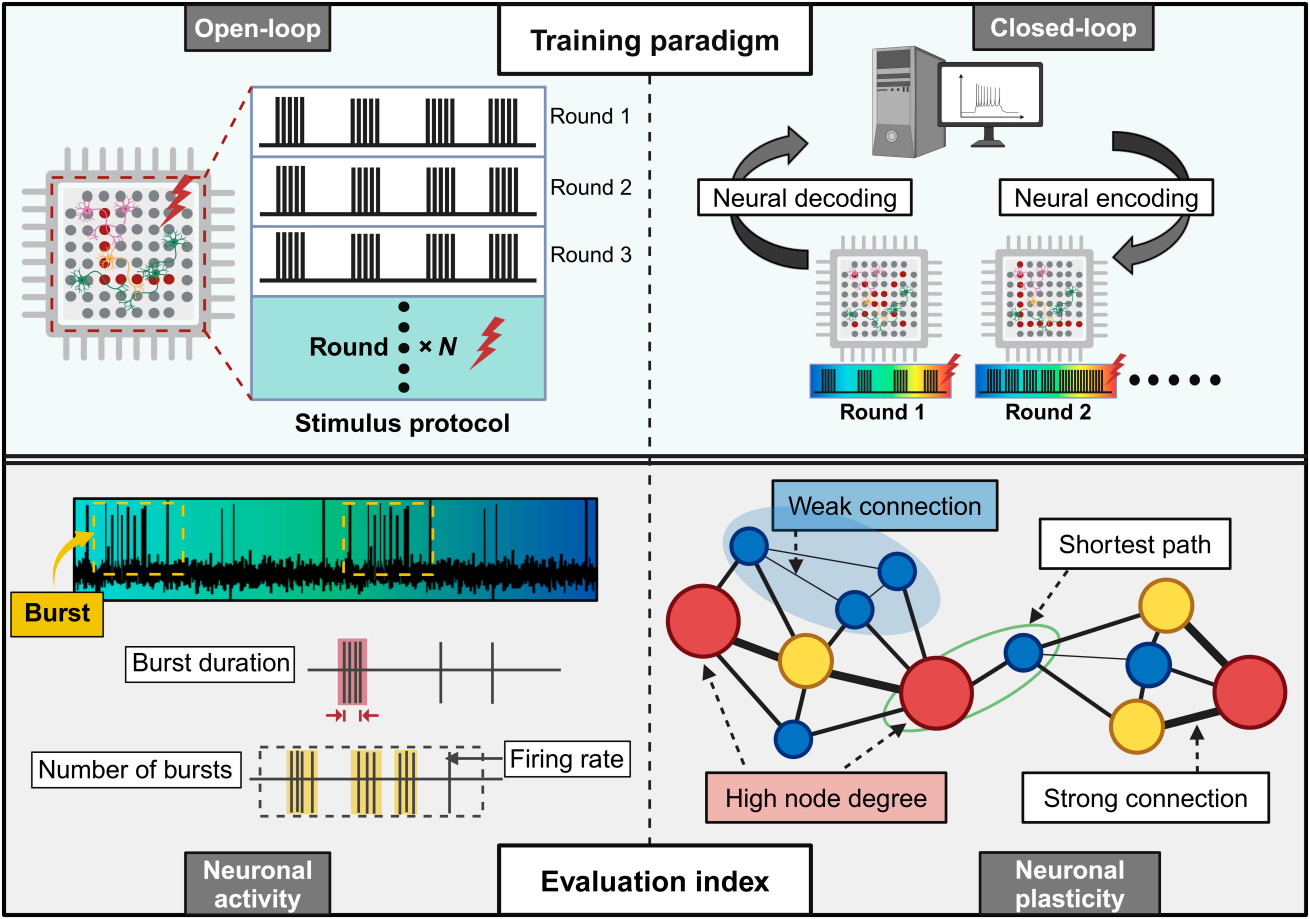
Training paradigm and evaluation index in BoCI systems. The training strategies for lab-grown brains can be classified into open-loop and closed-loop training. The effects of training on the biological neural network can be analyzed from 2 aspects: neuronal electrical activity and plasticity, which can be measured by functional network topology.

Neuronal network plasticity plays a fundamental role in the processes of learning and memory, achieved through dynamic synaptic weight adjustments in response to external stimuli. Electrical stimulation for inducing plasticity in neuronal networks can be divided into 2 types: open-loop and closed-loop training (Fig. 3). By recording the electrophysiological parameters of neural networks, such as spike firing rate and burst rate, researchers can analyze changes in the electrical activity of biological neural networks induced by training. In addition, by constructing functional connectivity networks and analyzing network topology, such as node degree, researchers could assess changes in the strength of neuronal connections before and after training (Fig. 3). Open-loop stimulation utilizes fixed, predetermined parameters that remain unchanged, regardless of neuronal responses. This method is widely used to modify network topology and strengthen neuronal connections. Jimbo et al. [66] showed multi-site stimulation reshapes information flow and neuronal dynamics, thereby reshaping functional connectivity within neuronal networks. However, the repetitive use of preset stimulation patterns in open-loop systems may be affected by uncontrolled spontaneous neuronal activity, potentially reducing the reliability of neuronal responses. Closed-loop stimulation, on the other hand, dynamically adjusts stimulation parameters based on real-time neuronal activity, generating optimal conditions for inducing plasticity. Shahaf and Marom first demonstrated that repetitive stimulation combined with reward-based training could achieve target neuronal response frequencies in cortical neurons. This suggests that regulating functional connectivity depends on stimulation-induced discharge activity, enabling stable spatial connectivity and desired responses within defined timeframes [67]. Wülfing et al. [68] employed reinforcement learning to establish a closed-loop paradigm, allowing neuronal firing rates to remain at target levels for extended periods. Integrating biological neuronal networks with external systems, such as robots or vehicles, has demonstrated enhanced performance through closed-loop training [69,70]. Biological neuronal networks have successfully controlled robots in obstacle-avoidance tasks and learned complex behaviors, such as playing ping-pong within minutes, suggesting that neuronal networks possess advanced learning and adaptation capabilities [12,16].

## Biocomputing Ability of BoCI Systems

Stimulation modalities provide diverse external inputs to lab-grown brains, eliciting nonlinear neuronal responses and modulating synaptic plasticity to enable mission capability. However, lab-grown brains lack robust feedback mechanisms compared to human or animal brains. To address this, integrating lab-grown brains with computers or robots establishes closed-loop control systems, enabling reciprocal feedback. This integration maps neural activity to machine behavior and uses environmental feedback or task performance to regulate neural activity, achieving intelligent machine control by the on-chip brain referring to the in vitro brain integrated with electrodes and a computer [12].

**Fig. 4.**
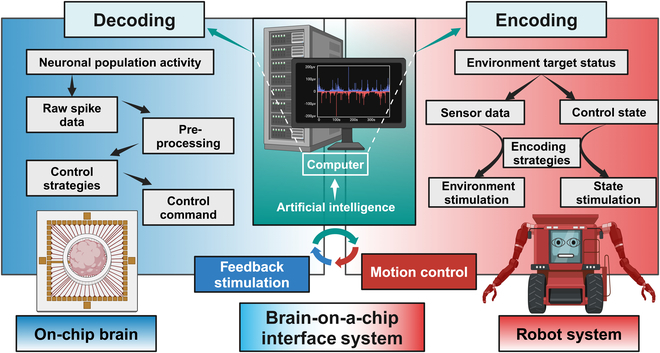
Brain-on-a-chip interface system for task execution. Bidirectional information interaction between the on-chip brain and the computer involves signal acquisition, where neural activity is decoded into control commands, and feedback stimulation, where environmental stimuli are encoded and applied to the neural network, while the interaction between the computer and robot includes robot control via decoded commands and feedback stimulation, with artificial intelligence (AI) algorithms optimizing coding and control strategies in the interaction process.

BoCI technology exemplifies the fusion of biotechnology and machines, leveraging nonlinear input–output mapping and synaptic plasticity. Through neural encoding and decoding, BoCIs enable real-time closed-loop information flow, allowing the on-chip brain to execute specific tasks [12]. A BoCI control system integrates cell cultures, neural signal interfaces, processing mechanisms, and machine control, forming a dynamic closed-loop control platform (Fig. 4). Current BoCI control systems consist of 3 subsystems: the on-chip brain system, the robot system, and the interface system. The on-chip brain system includes lab-grown brains, amplifiers, and stimulators to capture neural signals and deliver stimuli. The robot system, encompassing robotic or virtual systems, executes the on-chip brain’s decoded instructions and provides feedback on its operational status. The interface system bridges the above 2 subsystems, decoding neural activity and encoding input stimuli, with AI algorithms applied to optimize the encoding and decoding strategies within the interface system. Together, these components facilitate intelligent control and real-time interaction.

Different control tasks involving BoCI control systems can be categorized into 3 types: mobile robot control, robotic arm control, and virtual task (Table 1). Below, we discuss the integration of BoCIs and machines for each of these 3 control tasks.

**Table 1.** Tasks in BoCIs

Tasks	Methods	Mission
Mobile robot	Real-time encoding and decoding analysis of the electrical signals of lab-grown brains, which were regulated by electrical stimulation [71]	Robot to avoid obstacles in a circular area
	Closed-loop structure; reinforced learning method; lab-grown brains were controlled in real time by light and electrical stimulation [72]	Robot controlled to complete object-searching tasks in 2-dimensional space
	Electrical stimulation at 10 Hz; lab-grown brains were regulated according to the spike-timing dependent plasticity (STDP) of nerve cells [108,109]	Robot controlled to avoid hitting a wall
	Lab-grown brains were regulated by stimulation electrodes using a linear coding and decoding scheme and the reinforcement learning method [69]	Robot controlled to solve a maze
	By setting up a homeostasis-like property control system, lab-grown brains were regulated using both light and electrical stimulation [74]	
	By means of solutions containing different concentrations of magnesium ions, global or local chemical stimulation was performed in lab-grown brains [47]	Robot in a virtual environment to complete an obstacle-avoidance task
	By setting up a closed-loop system, depending on where the neurons fire in space in relation to the robot’s movement, 2 modes of electrical stimulation were used to regulate lab-grown brains [110]	Robot controlled to move in a direction
	By setting a closed-loop system, patterned training stimuli were applied according to the relationship between the spatial position of neuron firing and the robot’s movement, and lab-grown brains were regulated by electrical stimulation [111]	Robot controlled to move within a specified range
	Setting up a closed-loop system and electrically stimulating the brain based on the spatiotemporal pattern of neuronal spikes and the average activity of each output region [112]	Robot to avoid obstacles
Robotic arm	Closed-loop real-time recording and electrical stimulation to control brain activity on film [75]	Robot arm drawing
	Electronic stimulation to regulate the center of neural activity (CA) [113]	
	A closed-loop cybernetic system was formed through digital light signal input, the output of the robot arm, and control of lab-grown brains [76]	Camera-controlled robot arm to track an actual object
Virtual task	High-frequency or low-frequency electrical stimulation regulated the number of action potentials (APs) [16]	Program control to complete the arcade game “Pong”
	Applying the structure of closed-loop feedback for induced learning [77]	Aircraft vertical or horizontal flight control

### Mobile robot control

In various studies, on-chip brains have been used to control mobile robots for tasks like target tracking and obstacle avoidance. One study employed an on-chip brain to manage a robot’s navigation by sending sensor signals through MEAs that monitored the biological neural networks’ activity, which was then translated. This activity was translated into control signals for the robot [71]. Another experiment involved restructuring the neural network into 2 modules, enhancing the robot’s obstacle-avoidance capability by improving channel selectivity [69]. Additionally, a modular network was designed to control a robot’s movements based on sensory input, with synaptic weights modified through high-frequency stimuli, influencing the robot’s behavior [72]. Another research focused on decoding lab-grown brains’ response to different stimulus patterns, which were classified using artificial neural networks (ANNs), to regulate a robot’s movement direction [73]. In a different setup, lab-grown brains acted as computational libraries, training to guide robots toward targets. First-order reduced and controlled error learning was used to minimize discrepancies between output signals and target constants, with optogenetic feedback maintaining robot control [74].

### Robotic arm control

Bakkum et al. designed a closed-loop bio-robotic drawing machine, MEART, to explore network mechanisms for generating adaptive and goal-directed behaviors. They controlled the motion of the drawing machine’s robotic arm by measuring network activities, combining spatial and firing rate information that reflected cumulative changes in synaptic strengths. The control process involved comparing the robotic arm’s behavior to the desired behavior and then adjusting the training stimuli, which could change neuronal activity and subsequently alter the robotic arm’s movement and sensory feedback, creating a closed-loop system. This system enabled the robotic arm to draw in a desired direction [75]. Shultz et al. used digitized video signals as sensory inputs to lab-grown brains and combined them with a robotic arm as part of a closed-loop control system. The robotic arm was directly guided to move toward the target position within the input video [76].

### Virtual task

DeMarse et al. developed a simulated flight control system using on-chip brain, where synaptic weights within the neural network were adjusted through high-frequency electrical stimulation. By manipulating these weights in real time, based on flight trajectory data and feedback, the system controlled the simulated aircraft’s pitch and roll, optimizing flight stability and enabling the on-chip brain to guide the aircraft’s path [77]. In a separate study, Kagan et al. trained lab-grown brains to play the video game Pong using the free energy principle. They created a sensory-motor pathway linking the brain to the game environment, where the positions of the ball and paddle were encoded as stimulus signals. The neural activity in motor areas was recorded and used to determine paddle movement. Feedback was applied based on the brain’s performance: negative feedback was given for missed interceptions, while positive feedback was given for successful attempts. This setup allowed the on-chip brain to learn the game quickly [16]. Additionally, they discovered that structured sensory input crucial for task performance could drive the neural network toward a critical state, enhancing its ability to complete tasks efficiently. These findings highlight the potential of on-chip brains in real-time control systems and learning applications [17].

## BoCI-Based Hybrid Intelligence

BoCIs represent a substantial advancement in the field of hybrid intelligence. By leveraging BoCI technology, the intelligence of lab-grown brain networks can be harnessed and manifested in interactions with the external environment. This fusion of biological and artificial intelligence has the potential to profoundly impact research and development, offering new possibilities for advancements in both neuroscience and computational technologies.

AI uses algorithms, including ANNs, which consist of an input layer, hidden layers, and an output layer. By processing data and adjusting weights, ANNs can learn, reason, and process information. The physical reservoir computing model, a type of recurrent neural network, has 3 layers: input, reservoir, and output. External signals are processed in the reservoir, where information circulates among nodes, and the output layer decodes the response [9,74]. Unlike the artificial systems used in traditional reservoir computing (such as recurrent neural networks), BoCIs utilize biological neural networks, which inherently possess complexity, adaptability, and learning capabilities. This biological flexibility enables BoCI systems to process complex and dynamic signals. Biological neurons in BoCI systems exhibit neuroplasticity, meaning they can self-adjust in response to changes in input and environmental conditions [10,78]. This characteristic may make BoCI systems more data efficient in learning, reducing the need for large datasets. In contrast, traditional deep learning models require vast amounts of data and computational power to train effectively [79]. The power required for biological neurons to perform computations is far lower than that of silicon-based systems, allowing BoCIs to save energy during the learning process. Pizzi et al. explored stimulation-based learning with differentiated and undifferentiated cells, finding that differentiated cells exhibited specific responses, while undifferentiated cells showed random responses. This allowed ANNs to effectively decode and classify lab-grown brain response to stimuli [73]. Sumi et al. [9] investigated the role of cultured neural networks in reservoir computing, demonstrating that modular networks, created using micropatterned substrates, acted as generalization filters and achieved accurate classification in a spoken digit task. Furthermore, Yada et al. [74] highlighted the importance of homeostasis-like properties in lab-grown brains for solving complex problems. In another study as previously indicated [10], the “brainoware” system, using brain organoids as a reservoir, successfully performed tasks like speech recognition and nonlinear equation prediction. This system, trained with optical and electrical stimulation, benefited from unsupervised learning processes, enhancing its performance by modifying the organoids’ functional connectivity. These studies underscore the versatility and potential of lab-grown brains in advanced computational tasks within reservoir computing frameworks.

Additionally, other AI algorithms can be combined with lab-grown brains to create hybrid intelligence in some conditions. Kagan et al. employed the exponential-weight algorithm for exploration and exploitation (EXP3), an adaptive reinforcement learning algorithm. By dynamically adjusting synaptic weights based on real-time neural activity, the algorithm minimized the discrepancy between predicted and actual task performance, enabling rapid identification of the most task-relevant neural nodes. Using this approach, they successfully controlled lab-grown brains playing the “Pong” game in 5 min. The algorithm remains stable even with changes in inputs and does not undergo substantial mutations due to the real-time learning behavior of the lab-grown brains [16]. Buccelli et al. reconnected 1 of 2 neural modules separated due to intermediate lesions through a spiking neural network that simulates the spike signals of neurons in one module, thereby allowing for the regulation of neuronal activity in the other module. This provides a new technical tool for analysis and treatment of neuropathology, serving as a novel form of neuroprosthesis [80].

The optimal approach for modulating neural systems within lab-grown brains is facilitated by closed-loop systems. Chou et al. utilized a closed-loop architecture featuring an interface capable of both stimulating and recording neural network activity simultaneously, facilitating bidirectional information exchange between the lab-grown brains and ANN. Their investigation confirmed that closed-loop control yields superior efficacy in promoting functional development within lab-grown brains compared to open-loop control or individual neural network acting alone [81].

AI models based on physics principles play an important role in explaining the mechanisms of lab-grown brains. Bonifazi et al. [82] used a current-based silicon model to establish a mathematical model for small neural networks, explaining the dynamic characteristics of signal transmission between neural network synapses. Yamamoto et al. [83] employed an experiment-based silicon model to reveal that modularity is a functional advantage in the complex networks of lab-grown brains, where information is both separated and integrated between modules. Yamamoto et al. constructed a spiking neural network model based on leaky integrate-and-fire neurons to clarify the inherent mechanism by which lab-grown brains modularity enhances stability against external disturbances. They found that the modular architecture of a lab-grown brain plays a crucial role in resisting noise interference in asynchronous stimulation, and this effect is related to the persistent depletion mechanism of synaptic resources [84].

**Fig. 5.**
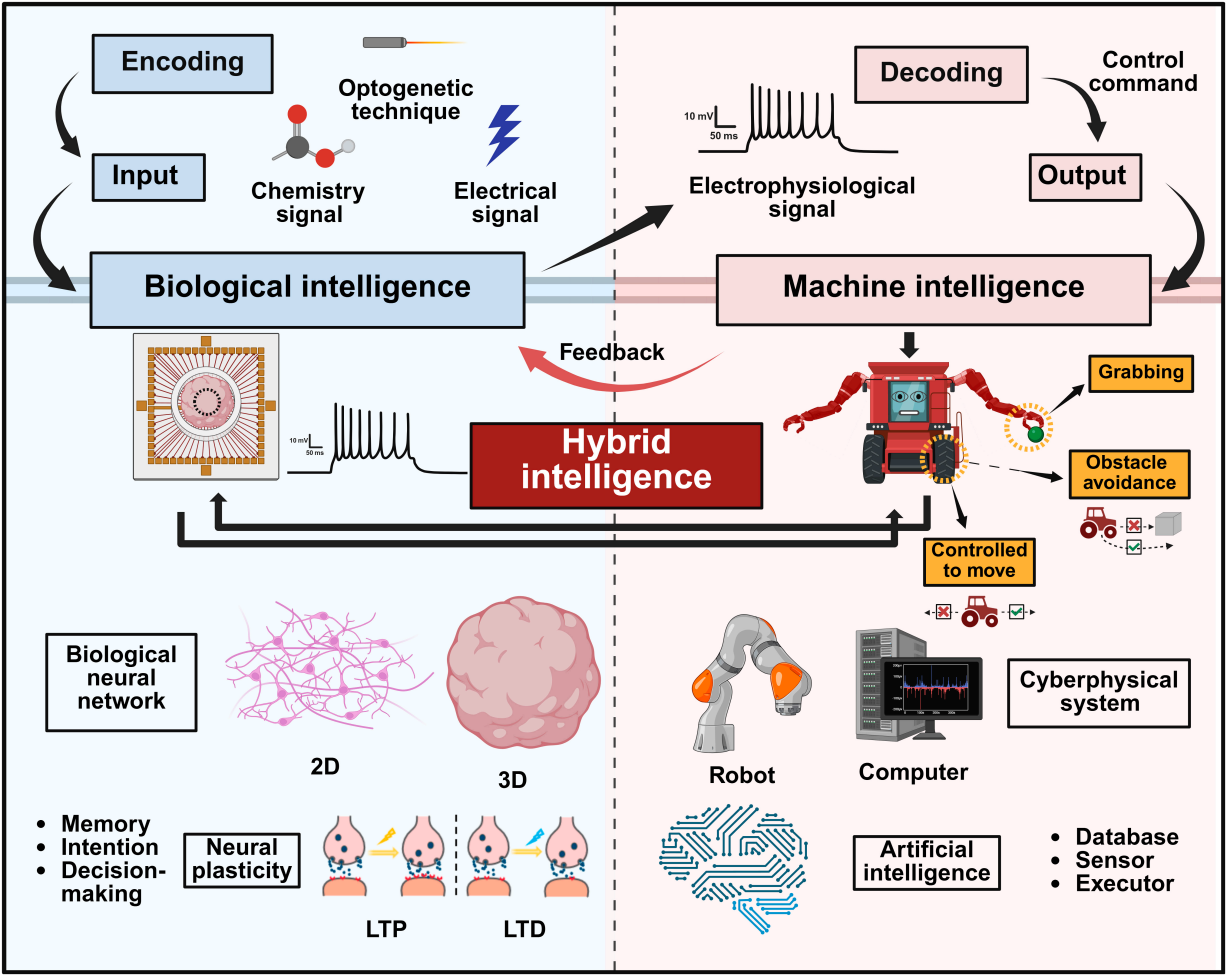
Hybrid intelligence framework based on a BoCI. In the BoCI system, biological intelligence is manifested through the learning and memory capabilities formed by 2-dimensional (2D) or 3D biological neural networks in response to input stimuli, driven by neural plasticity. These capabilities can be demonstrated through machine intelligence, such as AI-assisted decoding, to perform computer-mediated robotic tasks. LTP, long-term potentiation; LTD, long-term depression.

Notably, propelled by the evolution of 3D brain organoid technology, Thomas Hartung and his team [85,86] coined the term “organoid intelligence” (OI) to encapsulate the ongoing advancements in biological intelligence. Moreover, a collaborative initiative has been advanced to advancing OI research across multidisciplinary domains [87]. OI represents a multidisciplinary field focused on biocomputing through the use of brain organoids and brain–machine interface technologies, aiming to achieve hybrid intelligence [85,86]. Biocomputing systems based on OI are envisioned to tackle increasingly complex tasks with faster decision-making processes while exhibiting perpetual learning, high energy efficiency, and superior data conversion capabilities. Additionally, biological intelligence derived from human brain models holds great promise for elucidating the pathophysiology of neurodegenerative diseases like Alzheimer’s, paving the way for innovative therapeutic strategies [85–87]. A notable milestone in OI research was achieved in 2023, when Cai et al. [10] introduced the “brainoware” system, demonstrating the reservoir computing abilities of brain organoids in speech recognition via electrical stimulation. While OI primarily focuses on 3D brain organoids, its principles align with hybrid intelligence. Similarly, in BoCI systems, both 2D and 3D lab-grown brains exhibit memory, reservoir computing, and decision-making abilities. By combining these biological networks with machine intelligence (including computers, robotic devices, and AI), a novel hybrid intelligent system emerges, capable of data storage, perception, and decision execution (Fig. 5). This collaboration between biointelligence and AI heralds a transformative future for hybrid intelligence systems.

## Major Challenges and Future Trends

**Fig. 6.**
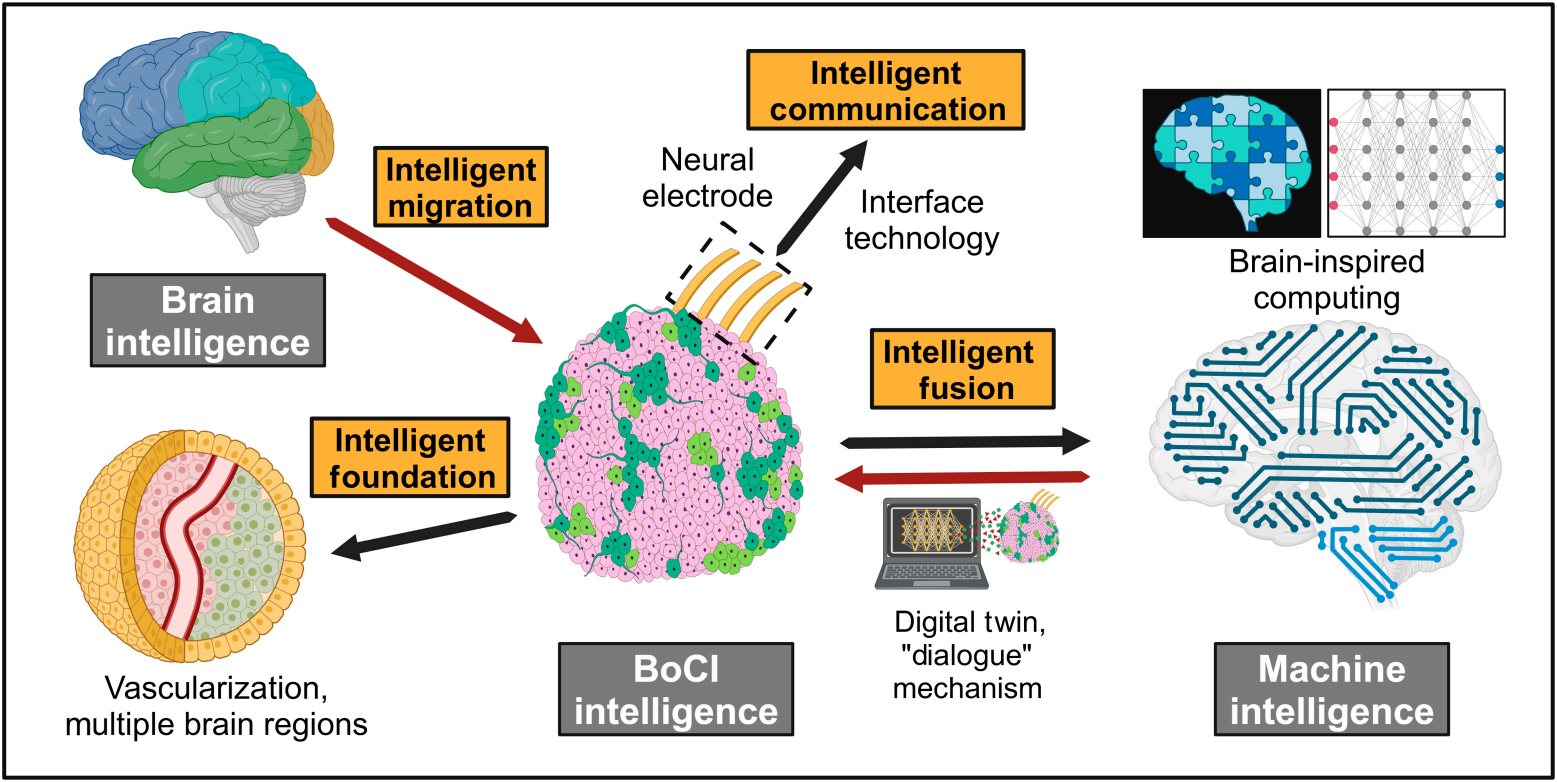
Challenges and future directions of BoCI systems.

The development of BoCIs is still in its early stages, facing substantial scientific challenges (Fig. 6 and Table 2). One key challenge is the establishment of a robust intelligence foundation. Currently, 2D neural networks are commonly used in BoCI systems, but their lack of spatial structure limits the complexity of neural connections and the number of neurons. In contrast, brain organoids with a 3D structure and complex neural connectivity have greater potential to simulate the human brain. However, these organoids still face limitations in terms of maturity, brain region specificity, size, and microenvironment. To overcome this, researchers are exploring models that fuse multiple brain regions to improve information transmission and processing [88–91]. Vascularization is particularly critical for ensuring oxygen and nutrient supply, which is necessary for organoids’ growth and maturation [92]. Without proper vascularization, organoids experience cell death in the inner regions, limiting their development. Converging disciplines—including synthetic biology and biomaterials—will further address these challenges. For example, ETV2-expressing cells contribute to forming a vascular-like network in cortical organoids [93], while co-culturing brain organoids with vascular endothelial cells enables self-assembly of primitive vasculature [94]. Additionally, the lack of proper sensory inputs and outputs hampers realistic brain development [95]. Research suggests that early neural activity, driven by sensory stimulation, is vital for functional neural circuit formation [96,97]. Therefore, stimulating brain organoids with multifactor inputs has been reported to accelerate maturation [48,98]. The maturation of cultivation techniques is crucial for obtaining stable brain organoids and serves as an important foundation for experimental reproducibility.

**Table 2.** BoCI development challenges and future directions

Section	Challenges	Priority	Directions
Intelligent foundation	Organoid maturity (3D structure, regional specificity)	High	Develop more mature 3D organoid models to enhance structural complexity and regional specificity
			Optimize vascularization technology to support organoid growth and stability
Intelligent communication	Electrode interaction optimization (rigidity of traditional electrodes compared to brain tissue)	High	Develop flexible optoelectrode interfaces to improve compatibility with brain tissue
Intelligent migration	Insufficient learning and adaptation capabilities in neural networks	Moderate	Improve training efficiency; design new training methods and coding strategies
	Lack of efficient training mechanisms; AI-assisted training is in its early stages		Optimize AI integration with organoid intelligence, applying deep learning models to enhance neural plasticity
Intelligent fusion	Difficulty in merging biological intelligence with AI (lack of effective encoding strategies)	Moderate	Deepen collaboration in hybrid intelligence to enhance machines’ understanding and responsiveness to biological networks
	Challenges in integrating AI with BoCI systems (incomplete learning mechanisms)		Promote AI and biological intelligence integration, developing cross-dataset decision support systems

The interface technology of BoCIs is crucial for enabling information exchange between lab-grown brains and external devices, forming the foundation for biological neural network and computer interfaces. The development of BoCI technology plays a key role in understanding the mechanisms of lab-grown brains’ intelligence and the generative theory of biological intelligence. Nowadays, the hardware interface in BoCI systems is based on MEAs. However, traditional electrode materials, such as silicon, carbon, and metals, are much stiffer than brain tissue, making them unsuitable for effective interaction [99]. As a result, there is a need for flexible neural electrodes with properties more similar to those of brain tissue. While flexible electrodes have been designed for in vivo applications [100], there is still a lack of electrodes tailored specifically for lab-grown brain systems [101,102]. Owing to their structural and mechanical compatibility with neural tissues, bioinspired flexible electronics have gained substantial attention as promising candidates for next-generation neural interfaces. Unlike rigid planar probes, these devices enable conformable 3D integration with brain organoids, facilitating long-term stable neural signal acquisition [31,35,36]. Future BoCI designs could synergize flexible electronics with organoid biomechanics to enhance functional fidelity. Additionally, current electrical interfaces face challenges like interference and stimulation-induced damage, hindering their effectiveness. Optoelectrode interfaces, which use light-based interaction, offer promising alternatives for improving brain–computer communication [103]. However, aligning the mechanical properties of optoelectrodes with those of brain tissue remains challenging. To advance lab-grown brain technology, the focus should be on developing high-throughput, flexible photoelectrode interfaces; ensuring long-term stability; and enabling the simultaneous recording of multiple brain regions for better performance and broader applications.

The engagement of lab-grown brains with the external environment can yield preliminary intelligent behaviors through information interactions [12], encompassing learning and memory. Applying electrical stimulation to lab-grown brains to emulate external information input facilitates information interaction with the external world, offering insights into the relationship between various neural networks and their collaborative operations [104]. Nevertheless, the absence of a learning mechanism for lab-grown brains’ learning currently hinders further improvement in its intelligent control capabilities. Current studies predominantly examine the effects of electrical stimulation on neural network discharge activity and topology from a global network perspective [105]. Enhancing the fine-grained understanding of neural network information processing mechanisms and improving learning and intelligence represent formidable challenges that need to be addressed. Analysis of in vivo brain motor control information reveals potential in abstracting computational rules from high-dimensional neural data, represented as low-dimensional manifolds in hidden spaces [106,107]. These low-dimensional manifolds may elucidate the fundamental dynamic characteristics of neural activities, thus aiding in the comprehension of neural activity dynamics [106]. Emulating in vivo computational neuroscience methods could help investigate learning mechanisms, and design regulatory strategies for lab-grown brain systems hold promise.

As mentioned above, a promising avenue involves the implementation of intelligent controls predicated upon lab-grown brain technology, establishing bidirectional interfaces with robots [12,16]. However, extant research endeavors in the domain of hybrid intelligence have predominantly centered on 2D neural networks, with limited exploration into brain organoids, exemplified by the scarce literature in this area. Nonetheless, the advent of biocomputing systems leveraging OI, as proposed by Hartung et al., heralds a trajectory toward the realization of computational frameworks transcending the velocity, efficacy, and prowess of conventional silicon-based and artificial intelligence paradigms. The primary aim is to develop OI-based biocomputing systems that accelerate decision-making across heterogeneous datasets. These systems could enable perpetual learning, improved energy efficiency, and enhanced data processing. Extending from OI, the overarching aspiration of hybrid intelligence system based on BoCIs is to instigate a paradigm shift within the realm of biocomputing, surmounting constraints associated with silicon-based computing and AI methodologies [85–87]. Several investigations have sought to integrate the biological intelligence inherent in lab-grown brains with AI algorithms, thereby creating hybrid intelligence systems capable of undertaking specific tasks. Thus, the key remaining challenge for BoCI systems is intelligence integration to achieve hybrid intelligence. Current research is hindered by a limited understanding of how lab-grown brains process external information and by the absence of effective encoding strategies for environmental inputs [12,72]. Training efficiency is also limited, and AI-assisted training remains in its early stages, without solid theoretical guidance for integrating AI with lab-grown brain intelligence [16]. This limits the learning mechanisms of lifelike robots and results in inefficient training. The integration of BoCIs with deep learning models is also a promising research direction, as biological computing systems inherently possess features such as adaptive learning and feedback loops, which are crucial for deep learning. Currently, deep learning models often require retraining from scratch when exposed to new tasks, whereas BoCI systems can leverage their biological neural networks to continuously update their internal state, reducing the costly retraining cycles. An effective integration of AI with lab-grown brain intelligence, leveraging advanced deep learning models [80], could enhance the brain’s plasticity, learning ability, and overall performance, advancing the field toward a novel era of hybrid intelligence.

The future trajectory of hybrid intelligence emphasizes the pursuit of deeper mutual cooperation, enhancing machines’ capacity for understanding, perception, and responsiveness to the needs of biological networks. Simultaneously, rooted in encoding technology, organisms will interact with intelligent systems with greater adaptability. This advancement is poised to deepen our understanding of neuronal information interactions, elucidate the principles of network architecture, and propel the development of cutting-edge technologies such as chip design and brain-like computing. Furthermore, it will accelerate the progress of in vitro brain organoids, fostering substantial advancements in neuroscience and revealing broader application possibilities.

Despite gradual progress in addressing the aforementioned technical challenges, prolonged in vitro cultivation of lab-grown brains within BoCI systems—accompanied by increasing structural and functional complexity, enhanced signal fidelity in input/output processing, and improved learning/memory capabilities—will inevitably introduce multifaceted ethical dilemmas. While BoCIs’ reliance on in vitro models mitigates certain ethical concerns inherent in in vivo studies, translational safety demands rigorous evaluation, particularly in terms of long-term biocompatibility, functional reliability, and standardized ethical frameworks. Resolving these challenges necessitates interdisciplinary collaboration among engineers, neuroscientists, and bioethicists to align technological innovation with societal accountability. A core ethical debate surrounding BoCI systems centers on whether they could engender entities with proto-consciousness or sentience potential. Within the broader OI field, Thomas Hartung’s team has advocated for embedded ethics—a proactive framework in which ethicists integrate with research teams to iteratively identify and mitigate ethical risks throughout the experimental lifecycle [84]. Given that BoCI development will confront stage-specific ethical quandaries, sustained engagement between ethicists and researchers is imperative to comprehensively address emerging moral implications.
